# Thyroid Cancer Persistence in Patients with Unreliable Thyroglobulin Measurement: Circulating microRNA as Candidate Alternative Biomarkers

**DOI:** 10.3390/cancers14225620

**Published:** 2022-11-16

**Authors:** Alfredo Campennì, M’hammed Aguennouz, Massimiliano Siracusa, Angela Alibrandi, Francesca Polito, Rosaria Oteri, Sergio Baldari, Rosaria Maddalena Ruggeri, Luca Giovanella

**Affiliations:** 1 Department of Biomedical and Dental Sciences and Morpho-Functional Imaging, Unit of Nuclear Medicine, University of Messina, 98125 Messina, Italy; 2 Department of Clinical and Experimental Medicine, Unit of Neurology and Neuromuscular Diseases, University of Messina, 98125 Messina, Italy; 3 Department of Economics, Unit of Statistical and Mathematical Sciences, University of Messina, 98125 Messina, Italy; 4 Department of Human Pathology DETEV, Unit of Endocrinology, University of Messina, 98125 Messina, Italy; 5 Clinic for Nuclear Medicine and Competence Centre for Thyroid Diseases, Imaging Institute of Southern Switzerland, Ente Ospedaliero Cantonale, 6500 Bellinzona, Switzerland; 6 Clinic for Nuclear Medicine, University Hospital, University of Zurich, 8091 Zurich, Switzerland

**Keywords:** serum miRNA, papillary thyroid cancer, thyroglobulin, anti-thyroglobulin antibody, excellent response, persistent disease

## Abstract

**Simple Summary:**

About 30% of patients with papillary thyroid cancer (PTC) experience persistent/recurrent disease within 10 years after an initial treatment, serum thyroglobulin (Tg) representing the gold standard for surveillance. However, the measurement of serum Tg is unreliable in the presence of anti-thyroglobulin antibodies (TgAb). The aim of this pilot study was to investigate the role of circulating miRNA as valuable biomarkers for the early detection of persistent disease in TgAb-positive PCT patients. In our series, the serum miRNA (221, 222, 375, 155, and 146b) levels were >two-folds higher in the PTC patients than the controls. Moreover, a decrease of 50% or more in circulating miRNAs levels compared to the baseline was observed in patients with an excellent response to therapy but not in those with persistent disease, respectively. Accordingly, serum miRNA kinetics may provide additional and independent information to early detect persistent disease in PTC patients with an uninformative Tg and emerge as a candidate alternative PTC biomarker.

**Abstract:**

Background: We aimed to evaluate the role of circulating miRNAs as a biomarker of the persistence of papillary thyroid cancer (PTC) in patients with an “uninformative” thyroglobulin (Tg) measurement. Methods: We prospectively enrolled 49 consecutive PTC patients with Tg-positive antibodies (TgAb) who had undergone a (near)-total thyroidectomy and ^131^I therapy (RIT). The serum thyroid stimulating hormone (TSH), Tg, and TgAb levels were measured before and at 6 and 12 months after RIT, respectively. The serum miRNA (221, 222, 375, 155, and 146b) levels were measured simultaneously. Results: The response to the initial therapy was assessed according to the 2015 ATA criteria. A decrease in 50% or more of serum miRNA over time was observed in 41/49 PTC patients, who showed an excellent response (ER), but six and two patients were classified to have an indeterminate/incomplete biochemical or incomplete structural response to initial therapy. Conclusion: Serum miRNA kinetics emerge as a promising biomarker for the early detection of a persistent disease in PTC patients with uninformative Tg results.

## 1. Introduction

Papillary thyroid cancer (PTC) represents the most common endocrine malignancy, and its incidence has been rising over the last few decades. Most patients diagnosed with PTC have an excellent prognosis, with the occurrence of 10-year survival rates being >90%. However, up to 30% of patients experience persistent or relapsing disease within 10 years after an initial treatment [1]. Therefore, the early identification of patients who are at a higher risk for persistent/recurrent disease is relevant. 

The measurement of serum thyroglobulin (Tg) is integral in the post-treatment monitoring of PTC [2,3,4,5,6,7]. However, the measurement of Tg is unreliable in the presence of anti-thyroglobulin antibodies (TgAb), and, more rarely, other interfering antibodies (i.e., heterophilic antibodies). Alternative methods for the measurement of Tg [i.e., a mass spectrometry Tg measurement, Tg (mini-)recovery, and the comparison between the Tg immunometric and radioimmunological measurement] have been proposed [8]. Currently, however, there are no methods for overcoming the interference of TgAb that offer both a sufficient accuracy and a sufficient sensitivity in clinical practice [9].

In such cases, the changes in the serum TgAb concentrations are used as an imprecise surrogate tumor marker. However, the TgAb concentrations do not precisely correlate with the tumor load, may fluctuate for non-specific reasons, and the remain detectable for years in some cases [8,9,10,11]. 

Recently, microRNA (miRNA) has emerged as potential disease biomarkers in different oncological settings, including differentiated thyroid cancer (DTC). miRNAs are small (about 20–25 nucleotides in length) noncoding RNAs involved in the post-transcriptional regulation of the gene expression. They inhibit the translation of target mRNAs into proteins and regulate about 90% of genes. Moreover, as a single miRNA may target up to several hundred mRNAs, an aberrant miRNA expression may affect a multitude of transcripts, deeply influencing the cell signaling pathways [12,13]. Thus, miRNAs are involved in the regulation of numerous biological processes (cell proliferation and differentiation, apoptosis, migration, metabolism, etc.) as well as in the onset and progression of several pathologies [12,13]. In particular, dysregulated miRNAs participate in the development and progression of cancer, acting as either oncogenes or tumor suppressors, and affecting crucial points in the cell cycle regulation, genome integrity, and metastatic spread [14,15,16]. All in all, miRNAs are promising disease biomarkers and, at the same time, putative therapeutic targets [17,18]. Recently, the altered expression of miRNAs was associated with the development of PTC and may represent a promising tool useful for its surveillance and prognosis evaluation [19,20,21]. In this light the potential role of circulating PTC-associated miRNAs has been preliminarily assessed with encouraging results [22,23,24,25,26,27].

The present study aimed to evaluate the role of serum miRNA as alternative tumor biomarkers for the early detection of persistent disease in TgAb-positive PCT patients with “uninformative” thyroglobulin values.

## 2. Materials and Methods

### 2.1. Patients

Three-hundred PTC patients (F = 205, M = 95, median age = 49 years; range: 20–75 years; female to male ratio = 2.7:1), who consecutively referred to the Department of Nuclear Medicine of the University Hospital “G. Martino” of Messina, Italy within 18 months following the approval of the study protocol, were considered as the initial cohort. The inclusion criteria for the study were: age >18 years, positive TgAb, papillary histotype, and an indication to radioiodine treatment. The exclusion criteria were: being of pediatric age, non-PTC histotypes (e.g., follicular and/or oncocytic histotypes), high risk patients according to the ATA risk classification, and the detection of metastases at the initial evaluation. 

On this basis, 49 TgAb-positive PTC patients (35 F, 14 M; F/M ratio = 2.50:1, median age = 48 years, range = 22–74) who gave permission to take a blood sample, were prospectively enrolled at the University Hospital of Messina, Italy. Based on the 2015 ATA risk classification [2], 30 patients had low-risk PTC (23 F, 7 M; F/M ratio = 3.28:1, mean age = 44.76 ± 11.54, median age = 46.5 years, range = 22–74) and 19 intermediate-risk PTC (12 F, 7 M; F/M ratio = 1.7:1, mean age = 47.68 ± 8.49, median age = 50 years, range = 29–57). As reported in the inclusion criteria of the study, all these patients were candidates to receive a radioiodine treatment because of a suboptimal surgery, multifocality/bilaterality, isthmic location of the malignant nodule. Two months after surgery, all patients underwent RIT with an ablative or adjuvant intent (2200–3700 MBq). A radioiodine treatment (RIT) was performed after a recombinant human-thyrotropin rhTSH-stimulation (standard protocol) using activities of 2220 MBq for the thyroid remnant ablation (low-risk PTC) and 3700 MBq for an adjuvant purpose. The serum TSH, Tg, and TgAb were measured both before and after the rhTSH stimulation. A post-therapy whole body scintigraphy (pT-WBS) was performed to assess the presence of thyroid remnant and/or extra-thyroid disease foci, as previously reported [28,29,30].

To evaluate the circulating microRNAs levels in PTC patients, venous blood samples were collected at the time of the RIT [basal evaluation, just before ^131^I (RAI) administration], 6 (first follow-up) and 12 (second follow-up) months after the RIT. For comparison, the serum samples were collected from 20 gender–age matched healthy individuals (14 F, 6 M; F/M ratio = 2.33:1, mean age 48.65 ± 6.82, median age = 50.5 years, range = 35–59), that served as controls (study entry sample). The 20 healthy controls (HC) were subjects who had been seen in the hospital’s outpatient clinics for periodic health assessments or complaints unrelated to the thyroid. All the HCs had no personal history of any cancer and no evidence of thyroid disease based on the results of a screening examination, including patient and family histories, neck ultrasound findings, and the results of thyroid hormone and thyroid antibody assays.

All subjects gave their informed consent for their inclusion before they participated in the study. The study was conducted in accordance with the Declaration of Helsinki, and the protocol was approved by the Ethics Committee of G. Martino” University Hospital (Project identification code 6420). 

### 2.2. Response Assessment and Follow-Up 

Six months after the RIT, a blood sample was obtained to assess the TSH and FT4 and adjust the thyroxine therapy. The response to the initial treatment was assessed 12 months after the initial therapy by the basal and stimulated-Tg and TgAb measurement, neck US, and diagnostic radioiodine whole body scintigraphy (Dx-WBS) and was classified as an excellent response (i.e.,: negative TgAb with undetectable Tg), an incomplete biochemical response (i.e.,: increase in Tg or TgAb values and negative imaging studies), an indeterminate biochemical response (i.e.,: stabile/mild reduction in TgAb values and indeterminate results of imaging studies), and incomplete structural response (i.e., the presence of structural disease detected by imaging studies and/or biopsy procedure), according to the 2015 ATA dynamic risk stratification system [2].

### 2.3. Imaging Studies

Neck ultrasonography (nUS) was performed in all patients at the first evaluation was by experienced operators using a high-resolution US scanner that was equipped with a high-frequency linear probe (14 MHz, General Electric Healthcare, Chicago, IL, USA). The thyroid bed and both the central and lateral lymph-nodes stations were systematically assessed. 

pT-WBS and Dx-WBS were performed 5–7 days and 2 days after the administration of 2220–3700 MBq or 185 MBq of ^131^I, respectively. The studies were obtained using a double-headed gamma camera (Millennium VG, GE Medical System. Chicago, IL, USA) equipped with high-energy low-resolution parallel-hole collimators (HEHRPAR). Whole-body images were obtained from the head to proximal thighs (anterior and posterior views, matrix 1024 × 256, magnification: 1, acquisition time: 10 cm/min). The study was integrated by static images of the neck and thorax (anterior and posterior views, magnification: 1; matrix: 256 × 256; frame time: 900 s). 

### 2.4. Laboratory Analysis

The sampled venous blood was collected in BD Vacutainer^®^ tubes with a separator gel and centrifuged at 3500 RPM for 15 min at 4 °C. The supernatant was isolated and centrifuged again to remove the circulating cells or debris. The serum aliquots were frozen and stored at −80 °C until the time of the analysis at the Central Laboratory, ‘‘G. Martino’’ University Hospital, Messina (Italy).

### 2.5. Biochemical Markers

The serum TSH, FT4, and TgAb were measured by an automated chemiluminescent immunometric assay (CLIA) on a UniCell^®^DxI automated platform (Beckman Coulter, Brea, CA, USA). The reference ranges were 0.3–4.2 mIU/L, 12–22 pm/L, and 0–4 IU/mL for TSH, FT4, and TgAb, respectively. The serum Tg was measured by an immunoradiometric assay (IRMA) (Cisbio Bioassays, Codolet, France) with an analytical and functional sensitivity of 0.2 ng/mL and 0.7 ng/mL, respectively. 

### 2.6. Serum miRNAs Measurement

Five miRNAs were selected for the serum measurement because their association with PTC is supported by substantial evidence in the literature [19,20]. They included miR-221, miR-222, miR-375, miR-155, and miR-146b, whose circulating levels and tissue expression have been reported to be significantly increased in PTC, with respect to the normal subjects/thyroid tissues in several studies [21,22,23,24,25,26,27]. In particular, miR-221 and miR-222 are the two most extensively evaluated functional miRNAs in thyroid carcinoma [31]. Increasing evidence indicates that miR-221, miR-222, and miR-146b are upregulated in PTC [24,27] and their expression significantly predicts the outcome and prognosis of the cancer [24,27,31,32,33,34,35,36].

#### 2.6.1. RNA Isolation, Reverse Transcription, and miRNAs Expression by Real Time PCR

Using a Qiagen miRNeasy Mini Kit (Qiagen, GmbH, Hilden, Germany), the total RNA was isolated from 800 μL serum samples, following the Qiagen Supplementary Protocol for the purification of RNA (including small RNAs). The RNA which was extracted was eluted in 200 μL of RNase-free water and was subsequently precipitated by adding 20 μg of glycogen, 0.1 volumes of 3 M of sodium acetate, and 2.5 volumes of ice cold 100% ethanol. After incubation at −80 °C overnight, the RNA was centrifuged and washed twice in ice cold 75% ethanol and resuspended in 7 μL of RNase-free water. The RNA was quantified by Nanodrop. The enriched miRNA fraction was converted to cDNA using the specific TaqMan MicroRNA Reverse Transcriptase kit (Life Technologies; Thermo Fisher Scientific, Inc., Waltham, MA, USA). 

#### 2.6.2. RT-Quantitative Polymerase Chain Reaction (qPCR) of miRNAs

The miRNA protocol quantification for each miRNA was performed on an AB-7300 RT-PCR system (Thermo Fisher Scientific, Inc., Waltham, MA, USA). Briefly, 2 µL of cDNA was used for each specific miRNA TaqMan assay according to the manufacturer’s instructions. All real-time (RT) reactions, including the no-template controls and RT controls, were performed in triplicate. RNU6 as a small nuclear RNA was used to normalize the miRNA expression levels owing to its known expression stability and its wide use as a loading control in several published miRNA expression studies. The relative fold expression and changes were calculated using the 2−ΔΔCt method [37]. The fold change in the expression of each miRNA observed in PTC patients in relation to the healthy subjects was determined by the mean of the 2−ΔΔC t values. The expression levels of miRNAs are indicated as a fold change. If the fold change equals 1, there is no up- or down-regulation. A fold change >2 or <0.5 suggested, respectively, an up-regulation or down-regulation in PTC patients versus the healthy controls (HC). A PTC/HC ratio >2 was considered to be suggestive of an altered expression of miRNAs in cancer patients. In the same manner, in PTC patients, a significant decrease in the serum miRNAs levels > 50% (i.e., a ratio < 0.5) at each point of the follow-up (six and twenty months) versus the baseline values was arbitrarily defined. 

## 3. Results

Thyroid remnants, but no extra-thyroid iodine-avid foci, were found at pT-WBS in all patients. The median preablation TgAb value was 201 IU/mL (range: 123–889) with a corresponding undetectable Tg (i.e., <0.7 ng/mL) in all cases. 

**Figure 1:**
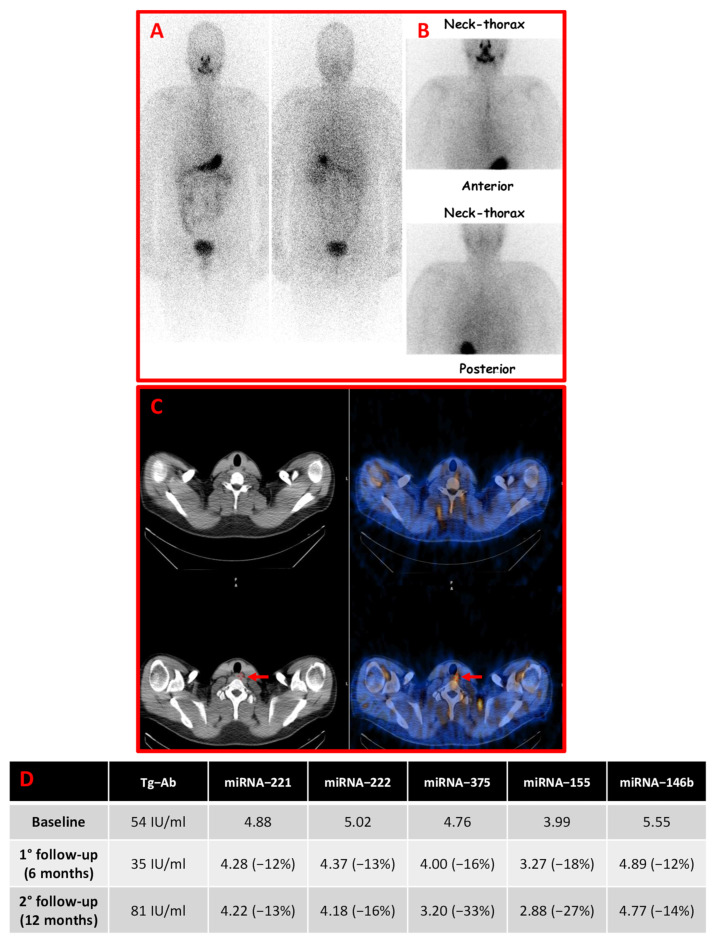
A male PTC patient, aged 54 years, with a multifocal and bilateral PTC [pT1b(m), Nx, Mx], and incomplete structural response (SIR) after initial treatments (surgery + RIT). Imaging findings and serum miRNAs changes are reported. Panel (**A**,**B**) ^123^I-Dx-WBS (222 MBq; anterior and posterior views) and planar imaging of the neck-thoracic region (anterior and posterior views). No abnormal radioiodine uptake was noted. Panel (**C**) SPECT/CT imaging (axial images) showed an abnormal radioiodine uptake involving at least a small-sized lymph node of the VI Robbins’ level (red arrows). (**D**) Changes in TgAb and circulating miRNAs 221, 222, and 146b levels during the follow-up in the patient.

The response to the initial treatment as assessed at 12 months was an excellent response (ER) in 41 cases, an incomplete structural response (SIR) in 2 cases (Figure 1), and an indeterminate or biochemical incomplete response (BIndR/BIR) in 6 cases. 

Among the latter, three patients had increasing TgAb levels (BIR), while in the remaining three patients, the TgAb levels remained stable over time (BIndR). Interestingly, the TgAb levels at the first follow-up (i.e., 6 months after RIT) were lower than the basal evaluation in all such patients.

At the basal evaluation, the serum miRNA levels were significantly higher in all PTC patients compared to the healthy controls, with a mean fold-change ranging from 3.11 to 3.64 (Table 1). 

Serum miRNA expression at different time points is reported in Table 2. 

**Figure 2:**
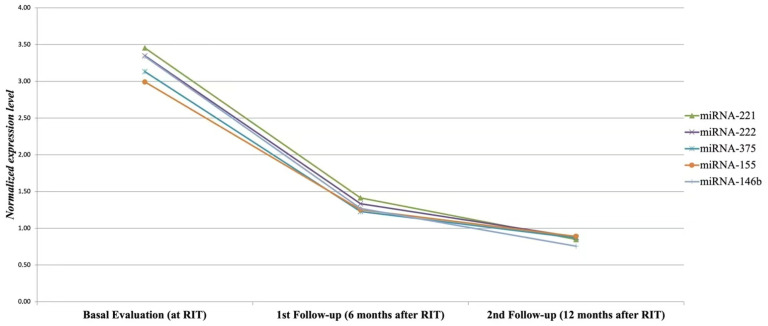
Serum miRNAs reduction rate between basal evaluation and first follow-up (≥50%) in patients with excellent response to initial therapy. Dynamic monitoring of circulating miRNAs, showing serum miRNAs reduction >50% compared to baseline during the follow-up in PTC patients with an excellent response to initial treatment (41 of 49 patients or 83.7%).

During the follow-up, the miRNAs levels decreased compared to the baseline in all PTC patients. However, a significant reduction in the serum post-therapy miRNAs levels (arbitrarily defined as >50% versus mean basal levels) was observed in 41 out of 49 patients (83.7%) at the first follow-up visit (6 months), with a further decrease at 12 months, that is in all patients with an ER to the initial treatment (41/41 or 100%) (Figure 2). 

**Figure 3:**
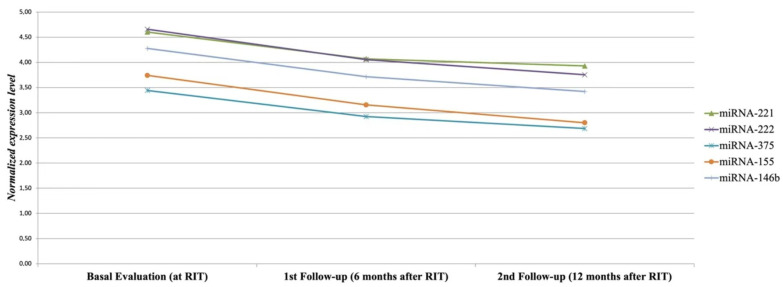
Serum miRNAs reduction rate between basal evaluation and first follow-up (<50%) in PTC patients with less than excellent (i.e., IndBR or ISR) response to initial therapy. Dynamic monitoring of circulating miRNAs, showing serum miRNAs reduction rates <50% during the follow-up in patients with BIndR/BIR or SIR (6 and 2 patients, respectively).

On the contrary, none of the eight patients without an ER (either BIndR/BIR or SIR) displayed a significant reduction in the post-therapy circulating miRNAs levels at 6 and 12 months, respectively (Figure 3). In more detail, miRNA miR-221, miR-222, and miR-146b showed the lowest reduction rates, predicting a non-ER with a 100% sensitivity and 100% specificity (Figure 3). 

It is noteworthy that the differences in the miRNAs kinetics emerged six months after the treatment and were not correlated with the initial decrease in TgAb observed in all patients. 

Finally, a receiver operating characteristic (ROC) curve was plotted using the miRNAs values of PTC patients (sensitivity) and HCs (specificity), but we were unsuccessful in finding optimal cut-off values able to detect PTC patients early without an ER likely due to the limited number of events (i.e., a non-ER). 

### Statistical Analysis

Continuous data were expressed as the mean, standard deviation, median value, minimum, and maximum. To compare the continuous variables in different patient groups, parametric and non-parametric tests were applied for normal and non-normal distribution variables, respectively. The Mann–Whitney test was applied to perform the comparisons, for all the examined numerical parameters, between PTC patients and the controls. Statistical analyses were performed using SPSS for Windows, version 22.0. A *p*-value < 0.05 was considered statistically significant.

## 4. Discussion

There is a rapidly expanding interest in evaluating the use of serum miRNAs as potential diagnostic and/or prognostic tumor markers. Indeed, these short, noncoding RNA molecules are involved in many biological processes, including cell proliferation and differentiation, and regulate the expression of several oncogenes and tumor suppressor genes. Thus, a dysregulated miRNA expression may play a role in the pathogenesis/progression of different malignancies [12,14,15,16,17,38,39,40]. Increasing evidence repeatedly demonstrated the key involvement of miRNAs in thyroid cancer, and an altered expression of tumor-associated miRNA has also been documented in thyroid cancer tissues [19,20,21,22,23,24,27]. Previous studies investigated the role of miRNAs (particularly miRNA 221, 222, and 146b) in differentiating between benign and malignant thyroid nodules and monitoring DTC patients [21,23,25,26,27,31,32,33,34,35,36,41,42,43,44,45,46] with sparse results, likely due to different methodological approaches to quantify the tissue expression of miRNAs. Recently, miRNAs have also been evaluated in body fluids like serum as they are quite stable being incorporated in microparticles [22,23,24,25,26,38,46], prompting us to evaluate their use as a circulating marker of disease persistence in PTC patients with “uninformative” thyroglobulin.

The present pilot study investigated changes in the serum levels of selected miRNA in a cohort of TgAb-positive PCT patients, in order to evaluate their potential role as reliable biomarkers of disease persistence. As the main result of our study, we found a significant association between a post-therapy decrease in miRNA levels of 50% or more at six months and an ER. Moreover, unchanged levels (i.e., <50%) correctly predicted disease persistence in patients with a TgAb positivity with a 100% sensitivity and 100% specificity. Overall, among the different serum miRNAs tested in our study, miRNA 221, 222, and 146b emerged as the most suitable candidate biomarkers to complement imaging (i.e., nUS, Dx-WBS) in the post-operative monitoring of PTC patients with uninformative Tg. 

This is well in line with some previous reports demonstrating the up-regulation of miR-222, miR-221, and miR-146b in PTC patients compared to those with benign nodules and controls [23,24,25,31,32,33,34,35,36,45]. Rosignolo et al. reported that the serum levels of circulating miR-221 were consistent with ATA responses to therapy in PTC patients, supporting their role in post-treatment monitoring [25]. Lee et al. demonstrated that the tumor expression of miR-222 and miR-146b was associated with the recurrence of PTC and that circulating miR-222 and miR-146b levels revealed the persistence of PTC [27]. Moreover, miRNA-146b is overexpressed in PTC and its overexpression positively correlates with a cancer invasion, in line with the role of such miRNA in prompting a cell migration and invasion. Thus, miR-146b may represent a candidate prognostic biomarker for tumor aggressiveness and outcomes [27]. More recently, Zang and co-workers performed a post-operative dynamic monitoring of miRNAs levels in a series of in 106 patients with PTC, and found that the serum levels of miR-222, miR-221, and miR-146b were significantly increased in PTC patients compared to controls and their expression was significantly associated with adverse prognostic variables (extra-thyroidal invasion, metastatic lymph node disease, etc.). In addition, miR-222, miR-221, and miR-146b were overexpressed in recurrent PTC patients compared to the non-recurrent PTC group, suggesting that changes in the miR-222, miR-221, and miR-146b levels may serve as prognostic and recurrent biomarkers for patients after surgery [46].

To the best of our knowledge, this is the first paper reporting data on the relationship between the serum miRNAs signature and response prediction in PTC patients with positive TgAb. All in all, circulating miRNAs, mainly miR-222, miR-221, and miR-146b, may represent non-invasive and effective biomarkers of recurrence in TgAb-positive patients, mainly in those with persistent positive TgAb after treatment. The need for improving the risk stratification and dealing with the problem of TgAb is clearly stated in the 2009 American Thyroid Association (ATA) guidelines, which suggested that these aspects as areas for ongoing research [11]. In this light, the measurement of serum miRNAs may help achieve these goals.

Some limitations of our study should be discussed, and the wide inter-subject variability of miRNA expression reduces the statistical power of our present data, thus conferring them a preliminary significance. Therefore, these preliminary encouraging results should be confirmed on a large series of patients, by using an absolute analysis of the miRNAs expression levels rather than fold-changes to compare the levels of miRNAs among patients.

Second, we did not collect samples before surgery and pre- versus post-operative comparisons were precluded. Third, using a group of subjects who had undergone surgery for thyroid disease could be more appropriate as the controls (i.e., the absence of the thyroid gland in both groups).

## 5. Conclusions

In conclusion, serum miRNA level monitoring may offer a promising tool for improving the stratification of the risk of recurrence in PTC patients with uninformative Tg. In our hands, if there is no significant drop in the miRNAs 221, 222, and 146b levels (i.e., their level remains above 50% of the basal levels), persistent disease should be suspected, prompting additional diagnostic studies (e.g., 123/131I-Dx-WBS). Moreover, it should be underlined that no change in the miRNAs levels represents an early signal comparing to standard Tg/antiTg measurements. However, the potential of these miRNAs as circulating biomarkers for the surveillance of PTC warrants further study on larger cohorts. Our preliminary data provide a backbone for further prospective, ideally multicenter, studies in DTC patients and a uninformative Tg measurement. 

## Figures and Tables

**Table 1:** Levels of the tested miRNAs in serum samples from the 49 PTC patients (at baseline) and 20 HCs (study entry sample).

	miRNA-221	miRNA-222	miRNA-375	miRNA-155	miRNA-146b
PTC patients (n = 49)					
Mean ± SD	3.64 ± 0.89	3.56 ± 0.91	3.18 ± 0.63	3.11 ± 0.66	3.49 ± 0.78
Median	3.47	3.15	3.22	3.05	3.26
Range	2.45–5.86	2.75–6.50	2.11–4.76	2.24–4.89	2.98–5.55
Healthy Controls (n = 20)					
Mean ± SD	0.85 ± 0.21	0.76 ± 0.27	0.74 ± 0.32	0.78 ± 0.28	0.75 ± 0.25
Median	0.89	0.83	0.82	0.86	0.84
Range	0.24–1.11	0.11–1.11	0.11–1.15	0.11–1.14	0.32–1.12
*p*-value *	0.0001	0.0001	0.0001	0.0001	0.0001

* The expression levels of miRNAs are indicated as fold expression (<0.5 downregulation and >2 upregulation) compared to healthy controls. A PTC/HC ratio > 2 was considered suggestive of altered miRNAs expression. Mann–Whitney test was applied to perform comparisons between PTC patients and healthy controls. A *p*-value < 0.05 was considered statistically significant.

**Table 2:** Serum miRNAs expression in our PTC patients at different time points.

miRNAs *	Basal Evaluation	1st Follow-up	2nd Follow-up
221 mean ± SD median value	3.64 ± 0.89 3.47	1.84 ± 1.1 1.46	1.35 ± 1.20 0.97
222 mean ± SD median value	3.56 ± 0.91 3.15	1.77 ± 1.11 1.34	1.34 ± 1.12 0.99
375 mean ± SD median value	3.18 ± 0.63 3.22	1.50 ± 0.72 1.22	1.16 ± 0.72 0.97
155 mean ± SD median value	3.11 ± 0.66 3.05	1.56 ± 0.82 1.22	1.20 ± 0.77 0.99
146b mean ± SD median value	3.49 ± 0.78 3.26	1.66 ± 0.99 1.22	1.19 ± 1.03 0.79

* Relative fold expression and changes were calculated using the 2−ΔΔC t method. Results were reported as fold change.

## Data Availability

The data that support the findings of this study are available on request from the corresponding author [A.C.].
